# Biomechanical Performances of Networked Polyethylene Glycol Diacrylate: Effect of Photoinitiator Concentration, Temperature, and Incubation Time

**DOI:** 10.1155/2016/3208312

**Published:** 2016-01-27

**Authors:** Morshed Khandaker, Albert Orock, Stefano Tarantini, Jeremiah White, Ozlem Yasar

**Affiliations:** ^1^Department of Engineering and Physics, University of Central Oklahoma, Edmond, OK 73034, USA; ^2^Department of Mechanical Engineering, New York City College of Technology, Brooklyn, NY 11201, USA

## Abstract

Nutrient conduit networks can be introduced within the Polyethylene Glycol Diacrylate (PEGDA) tissue construct to enable cells to survive in the scaffold. Nutrient conduit networks can be created on PEGDA by macrochannel to nanochannel fabrication techniques. Such networks can influence the mechanical and cell activities of PEGDA scaffold. There is no study conducted to evaluate the effect of nutrient conduit networks on the maximum tensile stress and cell activities of the tissue scaffold. The study aimed to explore the influence of the network architecture on the maximum tensile stress of PEGDA scaffold and compared with the nonnetworked PEGDA scaffold. Our study found that there are 1.78 and 2.23 times decrease of maximum tensile stress due to the introduction of nutrient conduit networks to the PEGDA scaffold at 23°C and 37°C temperature conditions, respectively. This study also found statistically significant effect of network architecture, PI concentration, temperature, and wait time on the maximum failure stress of PEGDA samples (*P* value < 0.05). Cell viability results demonstrated that networked PEGDA hydrogels possessed increased viability compared to nonnetworked and decreased viability with increased photoinitiator concentrations. The results of this study can be used for the design of PEGDA scaffold with macrosize nutrient conduit network channels.

## 1. Introduction

Tissue engineering is a new field that allows the combination of engineering, biology, and material methods for developing new techniques with potential to create tissues and organs [1]. The ability of networked three-dimensional structure to elicit altered cell behaviors, including cell adhesion, has raised heightened interest in the scaffold materials for various biomedical applications, including orthopedic repair and regeneration [2]. Cells* in vitro* usually do not reproduce in a three-dimensional fashion unless being allowed to grow on scaffolding. The scaffolds should have appropriate characteristics such as pore size, shape, and mechanical properties to enable cells to grow in every dimension. The cells have to be able to attach, migrate, proliferate, and differentiate into various organs on the scaffold. Several engineered tissue grafts have been developed for the reconstruction of the injured hard and soft tissues [3]. Yasar et al. [4] used Lindenmayer systems, an elegant fractal-based language algorithm framework, in designing vasculature networks that could potentially be incorporated in hydrogel scaffolds like PEGDA. The reason for using PEGDA over other materials is that PEGDA is 3D networked structures that can be manufactured easily to allow for the cell growth at higher depth using photolithograph process. Photolithography is a process which is commonly used in microfabrication to produce the desired scaffolds with a high level of detail and precision. It has been found to be a valid method to manufacture multiple-layer scaffolds for allowing the constructions of channels within the scaffold to better distribute nutrients to the cells. Yasar et al. [4] study also found that Polyethylene Glycol Diacrylate (PEGDA) tissue scaffolds having thickness higher than 1 mm were shown to have limited applications as a three-dimensional cell culture device due to the inability of cells to survive within the scaffolds. Without access to adequate nutrients, cells placed deep within the PEGDA tissue construct having thickness higher than 1 mm die out, leading to nonuniform tissue regeneration.

Photopolymerization system is usually comprised of three major parts: (1) a UV light source, (2) mold, and (3) a polymer solution. The role of the mold is to allow the PEGDA to polymerize in the desired shape. Tissue scaffolds, with nutrient conduit networks, need to be designed with intricate architecture, porosity, pore size and shape, and interconnectivity in order to provide the required structural strength, transport nutrients, and the microenvironment for cell and tissue ingrowth. By selecting the appropriate unit cell interior structures, structural properties such as the mechanical strength, ductility, and permeability and biological activities such as cell viability, degradation, and tissue generation of PEGDA structure can be controlled. The relationship between the interior nutrient conduit network structure and biomechanical properties (mechanical and biological properties) of PEGDA is not understood yet. Knowledge of the biomechanical properties of the networked PEGDA constructs with respect to the photoinitiator (PI) concentration, temperature, and incubation time is also necessary for adequate design and effective use of PEGDA for tissue engineering constructs.

To understand the effect of nutrient conduit networks on the PEGDA biomechanical performances, this study compared the failure stress of PEGDA flat dumbbell-shaped mold with nutrient conduit networks from the displacement controlled tension tests. Different photoinitiator concentration affects the materials properties due to the difference of crosslink density due to the difference of amount of free radicals created by the PI during the UV photopolymerization process. In addition, PEGDA hydrogels were susceptible to time and temperature dependent degradation, which in general negatively affect the mechanical strength. The failure assessments of the PEGDA molds were conducted in this study as a function of the PI concentration, temperature, and time. In addition, the cell viability assessments of with and without nutrient conduit networks cylindrical PEGDA molds were conducted as a function of the PI concentration. Flat dumbbell shaped PEGDA tension test samples with nutrient conduit networks at the gauge section were designed and fabricated by UV-photo polymerization process. PEGDA solution was added to different concentrations of PI (0.2% and 0.6%) solution to make the specimen. Tension tests were conducted on those samples at different temperatures (23°C and 37°C) and incubation time (0 and 7 days) after the fabrication of the specimen. Human DP147 mesenchymal fibroblasts cells were encapsulated within PEGDA hydrogel having nutrient conduit networks during the photopolymerization of the PEGDA. Cell viability experiments analyzed effects of conduit networks and photoinitiator concentration after 7 days of cell culture on the hydrogels.

## 2. Materials and Methods

### 2.1. Materials and Sample Types

Two solutions, PEGDA (*M*_*n*_ = 700; Sigma-Aldrich) with the Phosphate Buffer Solution (PBS) solvents, and the photoinitiator (PI), alpha-alpha-dimethoxy-alpha-phenylacetophenone (*M*_*w*_ = 256.35 g/mol; Sigma-Aldrich) with the 1-vinyl-2-pyrrolidone (*M*_*w*_ = 111.14 g/mol; Fluka) solvents, were used to fabricate the gel solutions. PBS was used instead of water in this study, since PBS is better biological solvent than water when preparing cell encapsulating PEGDA gel. Three samples were made for each experimental group of samples to evaluate the different concentrations of photoinitiator (0.2% and 0.6%) temperatures (23°C and 37°C) and incubation time (0 and 7 days) effect on the tension failure stresses of the samples. The reason for selecting 0.2% and 0.6% concentrations of photoinitiator for PEGDA solution was that in our earlier studies cells were successfully grown in PEGDA structures with significant cell viability differences with those concentrations of photoinitiator [5].

### 2.2. Mold Preparation to Prepare Mechanical Tests Samples

**Figure 1:**
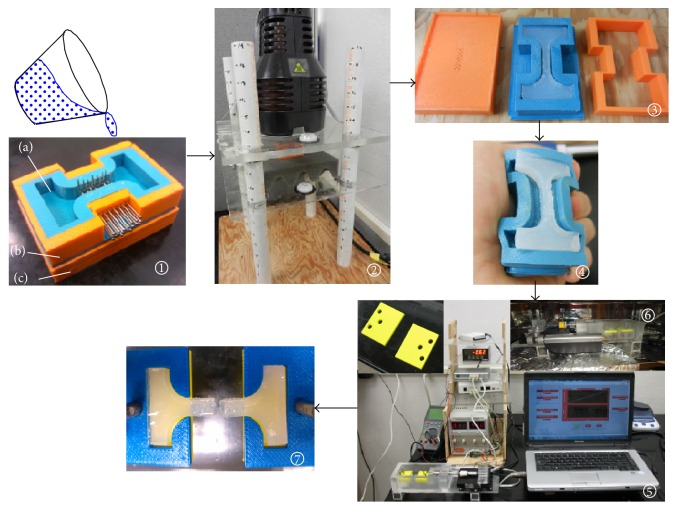
Steps that are performed for finding the failure stress of a networked PEGDA. Step 1: 20% PEGDA in PBS mixture was added to the desired concentration of photoinitiator mixture and poured in the custom-made mold to cure the mixture in flat dumbbell shape. Step 2: the solution was exposed to UV light for 3 min. Step 3: the mold was disassembled by the careful removal of pins. Step 4: the silicon mold was flexed to easily extract the specimen without damaging the PEGDA specimen. Step 5: tension test on PEGDA samples at room and incubator (body) conditions. Step 6: analysis of load and displacement data for the calculation of the failure stress of the specimen.

**Figure 2:**
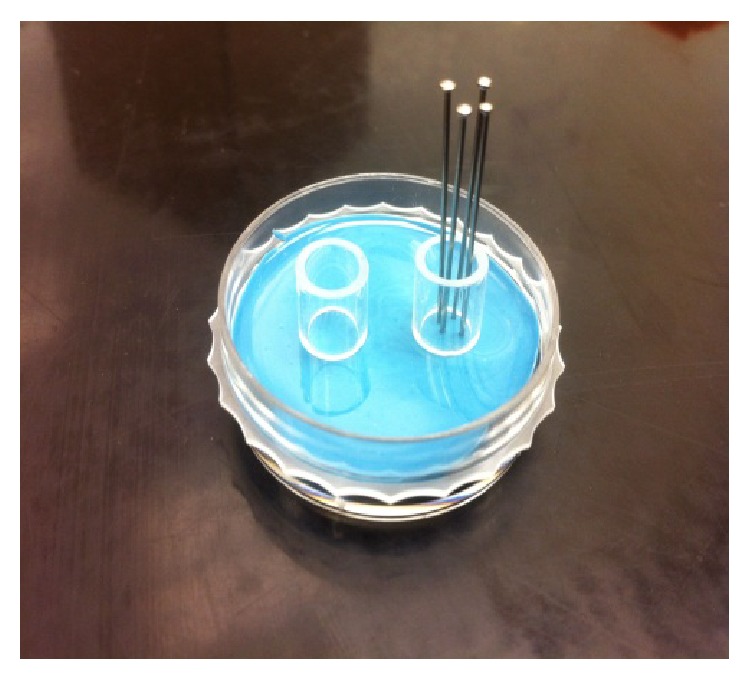
Networked and nonnetworked PEGDA hydrogels tissue culture dish with borosilicate glass hydrogel molds on silicon disc. PEGDA hydrogels with encapsulated cells were cured in the molds. Steel pins were inserted to create network conduit channels for networked PEGDA hydrogels (right), where nonnetworked PEGDA hydrogels curing was done without the presence of the pins (left).

Figure 1 shows the steps used for the preparation of the mold, specimen, and mechanical tests. A silicon (Casting Craft Easymold Silicone Rubber, Environmental Technology Inc. Fields Landing, CA) mold (Figure 1-*①*-(a)) was fabricated to make ASTM E855-90 standard [6] flat dumbbell-shaped for mechanical experiments. Two additional ABS plastic pieces (Figure 1-*①*-(b and c)), fabricated using Dimension Elite 3D Printer (Stratasys, Inc.), were assembled with the silicon mold to fabricate nutrient conduit networked PEGDA specimen. Three pieces of mold were used in this study to generate same nutrient conduit networked PEGDA specimen for all group of test samples, while only silicon mold was used to prepare PEGDA samples without nutrient conduit network. Each plastic piece has an array of holes (diameter: ~2 mm and spacing: ~1 mm). The bottom piece has 14 (7 × 2) holes, whereas the side piece has 21 (7 × 3) holes. A total amount of 35 pins (0.8 mm diameter) were inserted through these holes in the gauge section of the flat dumbbell-shaped mold as shown in Figure 1-*①*. The purpose of the pins is to produce an array of nutrient conduit network channels at the gauge section of the flat dumbbell-shaped PEGDA sample after UV polarization of PEGDA solution in the mold (Figure 1-*②*).

### 2.3. Mold Preparation to Prepare Cell Viability Tests Samples

An open-ended sterile cylindrical borosilicate glass tube was used to prepare mold for cell viability tests samples. One side of the open-ended tube was cured on silicon rubber disc to prepare cylindrical shaped hydrogels on the tube. The glass tube functioned as hydrogel housing as well as providing a support for pins to create nutrient architecture channels on hydrogel inside the tube. In addition, the glass tube provided a novel way to acquire thin section of hydrogel samples for viability assays. To create networked channeled PEGDA hydrogels thin stainless steel pins were carefully placed into the open glass end and secured into the silicon disc (Figure 2).

### 2.4. Mechanical Tests Samples Preparation

**Figure 3:**
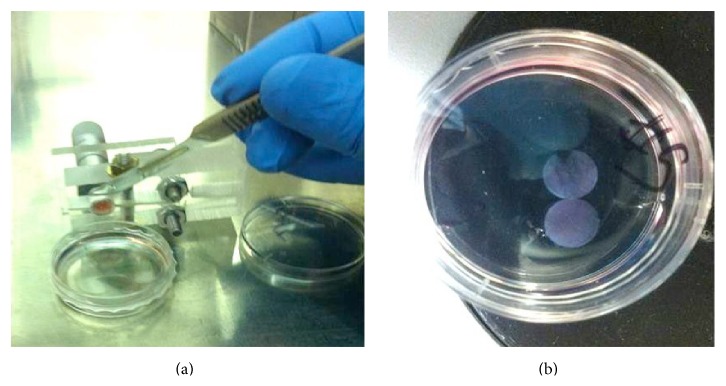
Sectioning of hydrogel specimen for cell viability experiments. (a) The cured cell infused hydrogel is placed in a custom-made holder equipped with micrometer. The micrometer allows for small increments of PEGDA hydrogel to be pushed outside of the tube to be sliced. (b) Thin hydrogel slices are obtained to be later analyzed under the microscope.

**Figure 4:**
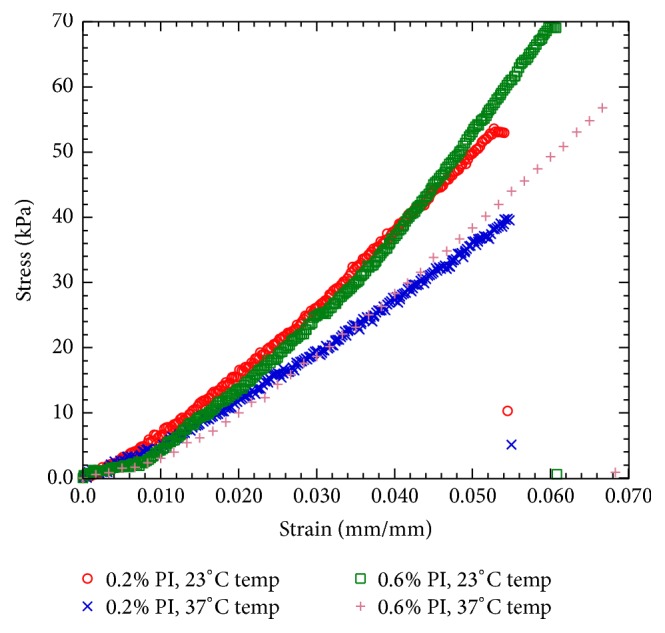
Typical stress versus strain diagram derived from the tension test on the flat dumbbell-shaped PEGDA sample having variable PI concentrations (0.2% and 0.6%) and test temperatures (23°C and 37°C). The tension tests were performed on these samples immediately after the preparation of the specimen at 0.01 mm/sec. strain rate.

The 20 wt% PEGDA solution was produced by mixing 2 mL of PEGDA with 8 mL of PBS. The PI solution was produced by mixing 0.3 g of PI with 1 mL of solvent in dark room to prevent premature crosslinking. The 0.2 wt% and 0.6 wt% PI gel solution was produced by mixing 4 *μ*L and 12 *μ*L of PI with 2 mL of PEGDA solution, respectively. The solution was poured in the custom-made mold to cure the mixture in a flat dumbbell-shaped gel. The solution was polymerized by exposure to 365 nm long wave UV (B-100SP Ultraviolet Lamp, UVP, LLC) light. In general, the biocompatibility of PEGDA hydrogel depends on complete polymerization of PEGDA solution while using minimal concentration of photoinitiator. The exposure to UV light causes photoinitiators to generate free radicals that initiate the polymerization to form the hydrogel. Since Mazzoccoli et al. [7] study found that 20 wt% and 40 wt% PEGDA having 0.6 wt% PI is biocompatible, therefore, this study used 0.2 wt% and 0.6 wt% PI gel solution to evaluate the PI concentration effect on failure stress of PEGDA samples. Due to the short-term UV exposure (3 to 5 minutes), photopolymerization is generally considered as a safe method to encapsulate cells [8]. Since encapsulation of cells is the main purpose of creating nutrient conduit networked in PEGDA gels, therefore, UV light was exposed for 3 min for all specimens to form the hydrogels and get the failure stresses of the samples. The pins were carefully removed from the solid plastic pieces after curing. The flexible silicon mold with hydrogel specimen was disassembled from the solid plastic pieces (Figure 1-*③*). The mold was bended to extract the PEGDA specimen (Figure 1-*④*). The specimen was stored in PBS solution before the mechanical tests. Eight groups of specimen were prepared.

### 2.5. Cell Viability Test Samples Preparation

DP147 dermal fibroblast cells, used in hydrogels, were acquired using standard techniques and protocols for culture and isolation. Cells used for culture were incubated at standard conditions, 37°C and 5% CO_2_, in tissue culture dishes with Dulbecco's Modified Eagle's medium (DMEM) containing 10% fetal bovine serum (FBS) and 1% antibacterial/antimicrobial (ABAM). Cells for hydrogels were isolated by removing nutrient media, washing with DPBS, and adding trypsin to break up cell tissues and suspend in media for counting. Suspended cells were counted three times with a hemocytometer and light microscope and averaged. After counting the average number of cells per volume the current population density was calculated. Cell suspensions were centrifuged for 5 minutes until cell pellets formed, separating cells from the media. Liquid was suctioned from cell pellet in test tube followed by adding the two hydrogel solutions and mixing thoroughly directly before UV curing [9].

Cultured human DP147 fibroblasts, for hydrogel seeding, were trypsinized and counted to add to hydrogel solutions. The desired hydrogel mixtures were added to the cell pellet and vortexed to ensure thorough mixing. Cell infused PEGDA solution was photopolymerized by UV light. Under the aseptic conditions of a biological safety cabinet hood, custom-made molds, for networked and nonnetworked hydrogels, shown in Figure 1 were placed in covered 35 (mm) tissue dishes for sterile curing. Hydrogel cell solutions were pipetted into a mold and cured in layers under the lamp. Excess liquid was removed under hood after each layer cured, and the following layers were added and photopolymerized. For networked hydrogels, caution was taken during removal of secured pins, not to damage the delicate structures. Finished hydrogel samples were rinsed twice in DPBS to remove the noncured hydrogel liquid solution. Next cured hydrogels were directly placed in new tissue culture dishes containing nutrient media and incubated at standard conditions of 37 degrees Celsius and 5% CO_2_. Nutrient media were removed and replenished every three days during incubation period.

### 2.6. Mechanical Tests

**Figure 5:**
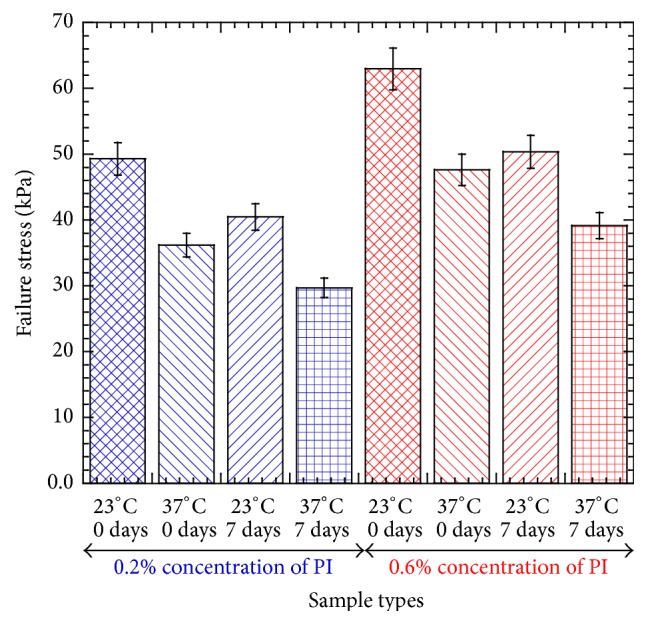
Tension test results of different PEGDA specimens showing the variation of the failure stress of the specimen due to photoinitiator (PI) concentration, incubation time, and temperature applied to the specimen during testing.

**Figure 6:**
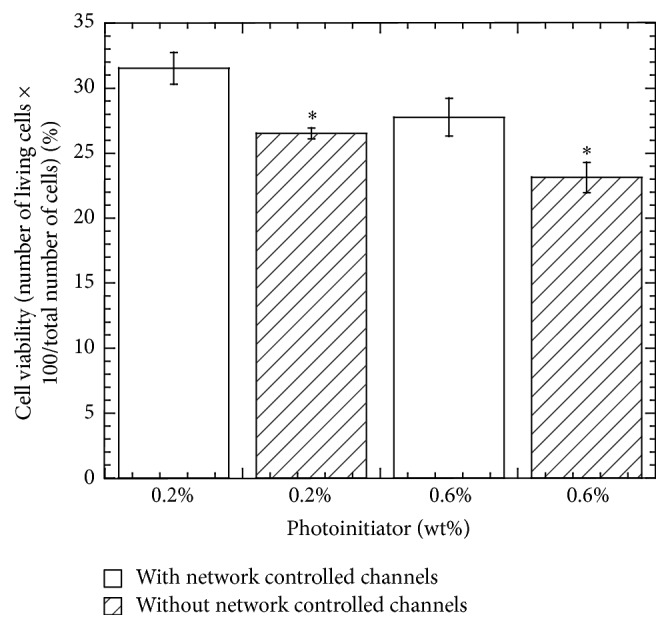
Cell viability test results of different PEGDA specimens showing the variation of the cell viability of the specimen due to the presence of network channel and photoinitiator (PI) concentrations of the specimen during testing.

A custom-made tension test setup was designed and fabricated for determining the tensile failure stresses of the specimens at room (laboratory) and physiological (NuAire NU-4750 incubator) temperatures. The complete test setup for conducting mechanical tests of PEGDA samples at room temperature is shown in Figure 1-*⑤*. The specimens were placed in the holders in an unstressed state. Cover plates, same sizes as the holders, were placed above the specimen to restrict upward movement of the specimen. A precision actuator (Newport™ LTA-HL^®^) was used in the setup to extend the specimens at a rate of 0.01 mm/sec until failure of specimen. Force was measured using 1 lb load cell (Futek™ LRM200) consistently throughout extension. Load cell was calibrated before testing. The force and displacement data were recorded simultaneously by a user written LabVIEW program 10.0 (National Instruments) from the load cell and actuator, respectively. For conducting mechanical tests of PEGDA samples at physiological temperatures (37°C), an electric connection was developed using the utility side access port of the incubator (NuAire NU-4750) to operate the actuator and load cell inside incubator (Figure 1-*⑥*), while doing data acquisition outside the incubator. A sample after failure is shown in Figure 1-*⑦*. The stress-strain curves were developed for each of the samples. Stress was calculated by the magnitude of the force divided by the cross-sectional area (~5.5 mm × ~7.5 mm) at the center of the gage length. Gage length (~15.7 mm) for the specimen was determined as the distance between the holders at the initiation of positive load to the specimen. Stress was calculated by the magnitude of the displacement after the initiation of load force divided by the gage length.

### 2.7. Cell Viability Assay

Viability of cells infused in PEGDA hydrogels was assessed using the Invitrogen LIVE/DEAD Viability/Cytotoxicity Kit, for mammalian cells (Molecular Probes, Invitrogen), and the fluorescent microscopy techniques. Two probes, calcein AM, and ethidium homodimer-1 (EthD-1) were used in the assay. Invitrogen fluorescence microscopy protocol for the LIVE/DEAD assay was followed. Optical filters were selected for optimum observance of calcein and EthD-1. Two different bandpass filters were chosen for the individual probes resulting in two fluorescent images, red and green. A digital camera, attached to the UV microscope, and computer imaging software captured, saved, and merged the (10x) magnified images. The final third image, combined red and green, consisted of two merged saved images. Viability was calculated from the merged images by counting the number of live green cells and dead red cells. The equation for hydrogel cell viability ((number of live cells) *∗* 100%/(number of live and dead cells)) was applied to each merged image. Multiple assay samples were collected from each hydrogel during culture to show the percent change in cell viability over the hydrogel incubation period.

After the curing, thin sample sections of the incubated hydrogel were collected for hydrogel cell viability at 7 days. Figures 3(a) and 3(b) show the sectioning of cell imbedded hydrogel specimen samples, enclosed in glass tube housing for separated slices by scalpel, for analysis of hydrogel cell viability. Thin even sections ranging from 0.5 to 1.0 (mm) were sampled from the incubated hydrogels with difficulty. Acquiring desired sample sections with certain dimensions for the viability without damaging the hydrogel structure was a difficult task. This process was improved by designing a prototype device (Figure 3(a)) to hold the hydrogel in the glass tube securely. Once the glass was secured the built-in micrometer could be turned to move the hydrogel out of the glass housing in desired increments for samples. A scalpel was used to section thin disks from the hydrogel. After sectioning the sampled hydrogel was rinsed with DPBS and placed back in normal incubation conditions. Sample sections were rinsed twice with DPBS to remove media. Next, the viability assay LIVE/DEAD solution was pipetted onto sample sections, in 35 (mm) tissue culture dishes. Sampled sections were covered with aluminum foil and incubated for 75 minutes. After the incubation period, sections were rinsed twice with DPBS, to remove excess stain, and placed on microscope slides that emerged in DPBS to prevent dehydration and remove unwanted liquids. Samples were assayed and viewed with two fluorescent microscope filters to produce an image for viability analysis.

### 2.8. Statistical Analysis

Statistical analyses were performed using Student's *t*-test for the different groups of specimen using Microsoft Excel 2000 statistical analysis toolkit. Datasets with a *P* value lower than 0.05 were considered significantly different.

## 3. Results and Discussion

Figure 4 compares the stress-strain curves between PEGDA hydrogels at variable PI concentrations (0.2% and 0.6%) and test temperatures (23°C and 37°C). The stress-strain response of all specimens is characterized as long elastic response, followed by a negligible inelastic region and then stable descending response. This result indicates that all PEGDA samples have brittle fracture behavior. This is reasonable since the conduit networks create three-dimensional voids on the samples.

Figure 5 and Table 1 showed a significant difference of maximum failure stress between the various PEGDA samples fabricated in this study due to photoinitiator (PI) concentration, incubation time, and temperature applied to the specimen during testing. This result clearly shows the higher PI concentration significantly increased the mechanical integrality of the PEGDA gel. This result can be explained with the fact that increasing PI concentration of the samples increased the crosslink density of the polymer matrix due to a larger number of reactive diacrylate groups, which, in turn, increased the failure stress of experimental samples. There is a thermodynamic relationship between the modulus (slope of stress and strain curve) and the crosslink density of a given polymer, where the modulus is directly proportional to changes in the crosslink density [10].

In each concentration, longer incubation (7 days versus 0 days) time and higher temperature (37°C versus 23°C) decreased the maximum failure stress of the PEGDA samples. This happens due to the fact that PEGDA undergoes small but significant degradation* in vitro* in PBS buffer solution as found by Xin et al. [11]. Such degradation lowers the mechanical integrity of PEGDA structures.

This study suggested that failure stress of PEGDA samples was highly dependent on the amount of the PI concentration and the methods by which it was processed. The failure stress of the nutrient conduit networked PEGDA samples is significantly lower than the natural liver tissue tensile stress of 232 kPa [12] and breaking stress of 451 kPa [13]. Higher concentration of PI can be used to increase the failure stress of the PEGDA based tissue engineering scaffolds as liver implant materials. In our earlier studies, it was found that cell viability was not as high in PEGDA scaffolds [5]. The cell viability was found higher for a concentration of PI of 0.2% than 0.6% for without network conduit PEGDA structure, whereas, in this study, it was found that higher concentration of photoinitiator contributes to toughening the hydrogels, increasing their failure strength. PEGDA tissue constructs had the strongest maximum failure strength when they are at room temperature compared to body temperature. Also PEGDA hydrogel was found to lose strength when tested a week after production under incubation conductions. This result was expected since PEGDA is a biodegradable material. More network conduit channels for cells to be exposed to media nutrient flow should increase the cell viability. The degradation of strength over time could be a result of other factors. The samples may have been exposed to high heat causing damage in the curing process during the fabrication of the scaffold. A device for thin samples needs to be implemented to get more accurate results. Variations on curing times, adding collagen, and cell seeding encapsulation prior to curing could improve the strength in addition as suggested to increase the cell viability [14].

Cells were successfully grown in UV crosslinked PEGDA hydrogel structures with significant viability differences in networked architecture compared to nonnetworked architecture PEGDA samples for both PEGDA gels having 0.2% and 0.6 wt% PI as shown in Figure 6. Statistical significant differences of cell viability between 0.2% and 0.6% PI concentration PEGDA samples were found after 7 days of incubation times for both channel and w/o channel PEGDA samples (*P* values < 0.05).

Viability results were not as high as expected when compared to 80% fibroblast viability at 14 days observed by other research [1], although network conduit channels exposing cells to nutrient flow demonstrated increased viability. The difference of cell viability results is due to the fact that PEGDA molecular weight and/or UV light intensity in the curing process was different from the previous authors. Variations on curing times and UV intensity, lowering of PI concentration, adding nutrients to hydrogel solution, increasing the cell seeding density, and substituting higher molecular weights of PEGDA could improve the cell viability of PEGDA hydrogel.

This study is limited to determine the effect of photoinitiator concentration, temperature, and incubation time on the mechanical and cell viability properties of network conduit channel PEGDA. The chemical properties (e.g., degree of polymerization and polymer spectra), surface characteristics (e.g., scanning electron microscope), and physical properties (e.g., swelling ratio, density) are beyond the scope of this study. The authors used same test setup and condition to prepare the different test samples. Since the sample preparation for different groups of sample is identical, therefore, the study assumes the chemical, surface, and physical properties of different test samples groups are identical.

In the future, microsize network conduit channel will be created on PEGDA by adding layered degradable fiber mat inside the PEGDA gel. The effect of photoinitiator concentration, temperature, and incubation time on the mechanical and cell viability properties on such produced PEGDA samples will be determined and will be compared with the macrosize network conduit channeled PEGDA samples results.

## 4. Conclusion

Tensile failure assessment of nutrient conduit networked PEGDA was conducted as a function of incubation time, test temperature, and photoinitiator concentration. This study concludes that the maximum failure stress of PEGDA can be increased significantly by the degree of photocrosslinking concentration, although significant decrease of failure stress occurs within 7 days of incubation time and at 37°C incubation temperature. Cell assay results demonstrated networked PEGDA hydrogels possessed increased viability compared to nonnetworked and decreased viability with increased photoinitiator concentrations. Further research using higher molecular weights of PEGDA, improved designs for networked molds, a device for attaining thin uniform assay samples, and the infusion of nutrients in hydrogels could increase the cell viability during incubation. Nutrient conduit networked PEGDA formed hydrogels can be tailored with adequate mechanical properties for various cell-based tissue engineering needs.

## Figures and Tables

**Table 1:** Statistical parameters determined from the tensile tests of different kinds of PEGDA samples with nutrient conduit networks.

Test conditions	A	B	C	D	E	F	G	H
Failure stress								
Number of samples	4	6	3	6	6	3	6	4
Average	49.30	36.17	40.45	29.68	62.95	47.64	50.35	39.12
St. dev.	4.24	2.40	2.30	3.37	5.63	3.03	3.90	1.75
*P* values								
Temperature effect		0.007 (AB)		0.004 (CD)		0.003 (EF)		0.001 (GH)
Incubation time effect	0.031 (AC)		0.007 (BD)		0.003 (EG)		0.039 (FH)	
Photoinitiator concentration effect					0.005 (AE)	0.017 (BF)	0.006 (CG)	0.001 (DH)

PEGDA samples with nutrient conduit networks are represented by letters A to H, where samples A have photoinitiator concentration = 0.2%, test temperature = 23°C, and incubation time = 0 days, samples B have photoinitiator concentration = 0.2%, test temperature = 37°C, and incubation time = 0 days, samples C have photoinitiator concentration = 0.2%, test temperature = 23°C, and incubation time = 7 days, samples D have photoinitiator concentration = 0.2%, test temperature = 37°C, and incubation time = 7 days, samples E have photoinitiator concentration = 0.6%, test temperature = 23°C, and incubation time = 0 days, samples F have photoinitiator concentration = 0.6%, test temperature = 37°C, and incubation time = 0 days, samples G have photoinitiator concentration = 0.6%, test temperature = 23°C, and incubation time = 7 days, and samples H have photoinitiator concentration = 0.6%, test temperature = 37°C, and incubation time = 7 days.

*P* values from the *t*-tests of failure stresses of two groups of specimen are represented by ( ).
